# Trap‐Programmable Ruddlesden–Popper Perovskite Nanocrystals for Persistent Optoelectronic Memory and Photonic Synapses

**DOI:** 10.1002/advs.78101

**Published:** 2026-09-29

**Authors:** Xuanyu Zhu, Qi Liu, Qi Wei, Hui Ren, Luwei Zhou, Jiangbo Peng, Yuxuan Chen, Jiatong Wang, Leyi Zhao, Pengzhi Wang, Xue Hao, Ting Wang, Mingjie Li

**Affiliations:** ^1^ Department of Physics and Materials The Hong Kong Polytechnic University Hong Kong China; ^2^ College of Materials and Chemistry & Chemical Engineering School of Nuclear Technology and Automation Engineering Nuclear Technology Key Laboratory of Earth Science Chengdu University of Technology Chengdu China; ^3^ Shenzhen Research Institute The Hong Kong Polytechnic University Shenzhen Guangdong China; ^4^ Photonics Research Institute The Hong Kong Polytechnic University Hong Kong China

**Keywords:** optical memory, persistent luminescence, photoexcited carriers, photonic synapses, Ruddlesden–Popper perovskites

## Abstract

Persistent luminescence offers a material‐native strategy for constructing non‐volatile optical memory. Nevertheless, embedding persistent luminescence functionality into solution‐processable optoelectronic platforms is still bottlenecked, as classic persistent phosphors suffer from poor film‐forming ability and low electrical conductivity for device integration. Herein, we report ligand‐free Sb‐doped Cs*_n_*_+1_Cd*_n_* Cl_3_ *_n_*_+1_ Ruddlesden–Popper perovskite nanocrystals (NCs), where the inorganic layer number (*n*) regulates local octahedral distortion and further tailors the intrinsic electron‐trap configuration. Benefiting from this precise structural modulation, the NC films achieve persistent luminescence with a duration over 5000 s and superior photoluminescence quantum efficiency. Combined spectroscopic characterization and first‐principles calculations reveal that the *n*‐tuned crystal framework generates differentiated trap energy levels, which dominate carrier trapping, storage and radiative release kinetics. Finally, a long‐lived ultraviolet‐sensitive photonic synapse is achieved by integrating optimized *n* = 2 perovskite NCs with single‐walled carbon nanotube networks. The fabricated device features ultralong persistent photocurrent, 160% paired‐pulse facilitation ratio, slow charge decay dynamics, and visual image retention capability. This work establishes a scalable solution‐processed perovskite platform bridging structure‐customized trap states, ultralong‐lived optical memory, and optoelectronic synaptic behaviors, which paves a feasible pathway for developing integrated sensing‐memory neuromorphic architectures.

## Graphical abstract

This work presents trap‐programmable Ruddlesden–Popper perovskite nanocrystals for ultralong‐lived optical memory and photonic synapses. Layer‐controlled octahedral distortion modulates trap depth and carrier‐storage kinetics, enabling persistent luminescence beyond 5000 s at room temperature. Integration with SWCNTs yields UV‐sensitive photonic synapses with persistent photocurrent, paired‐pulse facilitation, and pattern‐retention capability.

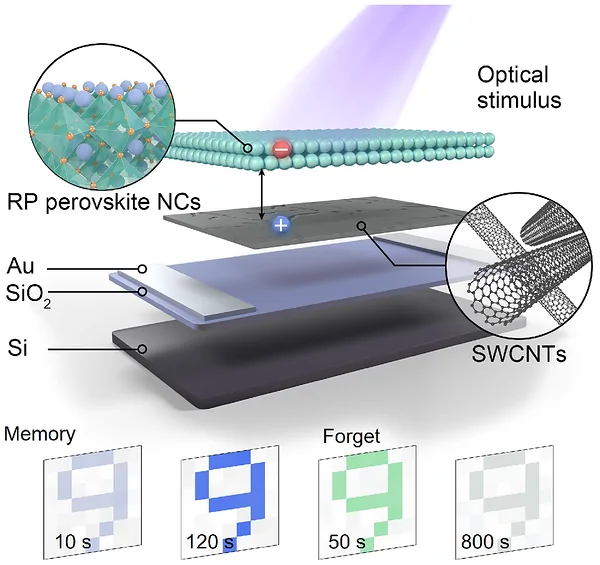

## 1 Introduction

The rapid growth of artificial intelligence, distributed sensing, and data‐intensive edge technologies has intensified the search for hardware platforms that can overcome the bandwidth and energy penalties of conventional von Neumann architectures [1, 2, 3, 4]. In biological systems, perception, memory formation, and signal processing are tightly coupled in space and time, allowing complex environmental information to be converted into adaptive responses with high efficiency [5, 6, 7, 8]. This principle has motivated the development of neuromorphic optoelectronic devices in which light serves not only as an external stimulus but also as a means of encoding temporal information [9, 10, 11]. Within this broader context, optoelectronic synapses are particularly attractive because they combine remote, low‐cross‐talk optical input with electronically readable output, thereby offering a route toward compact hardware that can sense, retain, and respond to optical signals in a unified element [12, 13, 14].

A key requirement for such devices is the availability of materials that possess intrinsic optical memory, namely the ability to store photoexcited carriers and release them over long timescales after illumination has ceased. Persistent luminescent materials naturally satisfy this criterion because excitation can be captured by metastable trap states and gradually released to regenerate delayed emission [15, 16, 17, 18]. Existing persistent optoelectronic memory systems are broadly classified into two types: one based on deep defect traps, which enable charge storage but provide no optical feedback; the other based on self‐trapped exciton (STE) emission, which offers intrinsic optical readout yet suffers from short carrier lifetimes due to rapid radiative recombination [19, 20, 21]. Conventional persistent phosphors have therefore provided a useful conceptual analogue to long‐lived biological memory [22, 23, 24, 25]. However, most classical inorganic systems rely on high‐temperature solid‐state synthesis, are difficult to process into uniform thin films, and often require insulating binders or matrices when incorporated into devices [26, 27]. These features limit charge transport, complicate electrical readout, and hinder the realization of scalable, solution‐processed photonic synapses based on persistent optical storage. As a result, there remains a significant materials gap between high‐performance afterglow phosphors and functional optoelectronic memory devices.

Halide perovskites are promising candidates for this challenge owing to their low‐temperature solution processability and broad compositional/structural tunability, and their optoelectronic devices have been widely explored for artificial vision and in‐sensor computing [28, 29, 30, 31]. Persistent luminescence has been demonstrated in several bulk or microcrystalline perovskite systems, but extension to nanocrystals (NCs) remains nontrivial because surface‐associated non‐radiative pathways become increasingly severe as particle size decreases [32, 33, 34, 35, 36]. At the same time, the nanoscale format is highly desirable for thin‐film integration, heterogeneous device fabrication, and spatially resolved optoelectronic function [37, 38, 39]. Ruddlesden–Popper (RP) halide perovskites are especially attractive in this regard because the inorganic layer number offers a structurally well‐defined handle for modulating local coordination, lattice distortion, and defect energetics. In principle, this framework should allow the temporal response of optical memory to be adjusted at the level of crystal chemistry rather than by empirical defect engineering alone.

Here, we develop ligand‐free, Sb‐doped Cs*_n_*_+1_Cd*_n_* Cl_3_ *_n_*_+1_ RP perovskite NCs that exhibit tunable room‐temperature persistent luminescence, which enables the development of ultralong‐lived optoelectronic memory devices. In this system, the Cs^+^ ion serves as an inorganic spacer, thereby eliminating the need for organic cations and avoiding their inherent instability [40, 41]. We demonstrate that the RP layer number directly modulates the distortion of the local [SbCl_6_]^3−^ coordination environment and thereby governs the dominant trap landscape. Spectroscopic measurements and first‐principles calculations consistently indicate that the *n* = 1 and *n* = 2 frameworks favor different carrier‐storage pathways, enabling layer‐dependent control over trap depth, detrapping kinetics, and optical‐memory duration. By integrating the optimized NCs with semiconducting single‐walled carbon nanotube (SWCNT) networks, we achieve UV‐sensitive photonic synapses with persistent photocurrent, paired‐pulse facilitation (PPF), learning‐forgetting‐relearning behavior, and image‐pattern retention. The resulting materials‐to‐device strategy shows how crystallographic control of trap states can be used to couple long‐lived optical memory with optoelectronic synaptic function in a solution‐processed platform.

## 2 Results

### 2.1 Synthesis and Layer‐Dependent Distortion

Ligand‐free, Sb^3+^‐doped RP perovskite NCs, specifically Cs_2_CdCl_4_:0.07Sb^3+^ (*n* = 1:Sb) and Cs_3_Cd_2_Cl_7_:0.07Sb^3+^ (*n* = 2:Sb), were synthesized through a modified one‐step injection process (Figure S1). Stoichiometric precursor solutions in dimethyl sulfoxide were rapidly injected into precooled isopropyl alcohol, triggering instantaneous nucleation of colloidal NCs. In the cross‐sections, the thickness of the RP perovskite films remained uniform at ∼1.1–1.3 µm (Figure S2). The ligand‐free character of this route is important for device integration because it avoids long insulating surface ligands that would otherwise suppress interparticle transport and interfacial charge exchange. Electron microscopy confirms the formation of well‐defined crystalline nanodomains with characteristic sizes of about 10–15 nm, while the observed lattice fringes match the expected RP phases. The NCs tend to assemble into compact aggregates rather than remaining as isolated particles, which is consistent with the absence of long organic ligands and is favorable for forming continuous solid films for optoelectronic measurements (Figure 1a). High‐angle annular dark‐field scanning TEM (HAADF‐STEM) and energy‐dispersive X‐ray spectroscopy (EDS) elemental mapping confirmed the homogeneous distribution of Cs, Cd, Cl and Sb throughout the NCs, indicating successful uniform doping (Figure S3).

**FIGURE 1:**
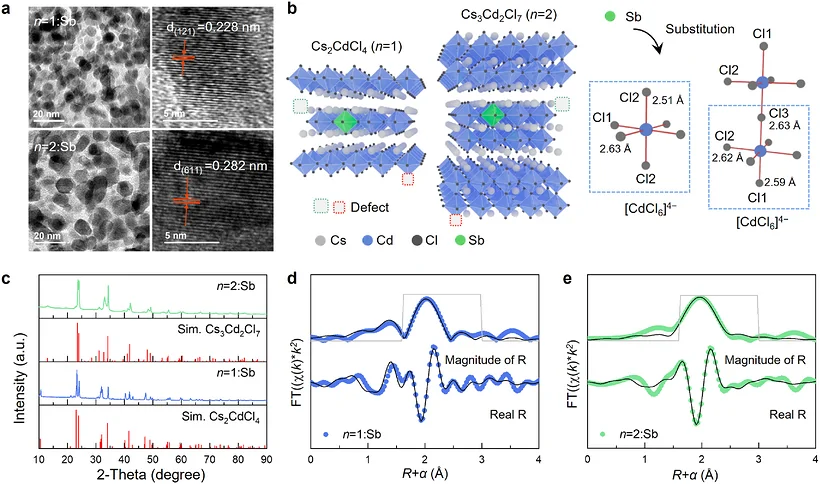
Microstructure of Sb‐doped Sb‐doped Cs*_n_*_+1_Cd*_n_* Cl_3_ *_n_*_+1_ RP perovskite NCs. (a) TEM and HRTEM images of *n* = 1:0.07Sb and *n* = 2:0.07Sb NCs. (b) Schematic crystal structure of RP perovskites (*n* = 1, 2), illustrating selective Sb substitution at Cd sites to modulate local defects and carrier trapping. (c) XRD patterns of *n* = 1:0.07Sb and *n* = 2:0.07Sb NCs. Sb K‐edge EXAFS data fitting in R‐space (real and magnitude) for (d) *n* = 1:0.07Sb and (e) *n* = 2:0.07Sb NCs. Fitting was performed with k^2^ weighting over an R‐range of 1.6–3 Å (gray lines).

Both host compounds adopt the tetragonal I4/mmm RP framework built from corner‐sharing [CdCl_6_]^4−^ octahedral slabs separated by Cs^+^ ions (Figure 1b). X‐ray diffraction (XRD) patterns (Figure 1c and Figure S4) confirm the phase purity of all samples. Rietveld refinement (Figure S5 and Table S1) quantitatively reveals systematic decreases in lattice parameters and unit cell volumes upon Sb incorporation, consistent with the substitution of Cd^2+^ by the smaller Sb^3+^ cation. This assignment is further supported by first‐principles calculations (Table S2), which indicate that occupation of Cd sites is energetically preferred over incorporation at Cs positions. Inductively coupled plasma optical emission spectrometry (ICP‐OES) analysis confirms Sb incorporation efficiencies of 6.65% and 6.16% for *n* = 1:Sb and *n* = 2:Sb, respectively (Table S3), closely matching the nominal 7% doping concentration. The slight difference in incorporation efficiency reflects the distinct Cd‐poor (*n* = 1) and Cd‐rich (*n* = 2) synthesis conditions, which affect competitive Sb^3+^ occupation at Cd^2+^ sites. Raman spectroscopy shows the host lattice modes (E_1g_, E_2g_, A_1g_) with no new modes upon doping, indicating that Sb incorporation does not disrupt long‐range crystal symmetry (Figure S6).^27^ X‐ray photoelectron spectroscopy (XPS) confirms the trivalent state of Sb (Figure S7). The results therefore establish that Sb activation can be introduced without disrupting the RP structural motif that underpins the layer‐dependent behavior of the system. The preservation of phase purity after heterovalent doping is especially important because it allows the subsequent trap and photophysical analysis to be interpreted in terms of framework‐controlled local structure rather than secondary phases or parasitic impurity emission.

To identify the local structural origin of the distinct memory behavior, we performed Sb K‐edge extended X‐ray absorption fine structure (EXAFS) measurements (Figure 1d,e). The analysis reveals a clear dependence of the [SbCl_6_]^3−^ coordination geometry on RP layer number (Figure S8 and Table S4). In *n* = 1:Sb, the activator environment is characterized by a relatively symmetric axial compression, whereas *n* = 2:Sb exhibits a more asymmetric distortion with one significantly shortened Sb–Cl bond. This difference in local geometry indicates that dimensionality not only alters the average crystal structure but also modifies the local crystal field experienced by the emissive center. As shown below, this layer‐dependent octahedral distortion provides the structural basis for the different trap landscapes, carrier‐release kinetics, and persistent‐luminescence lifetimes of the two NC systems. In this sense, the RP layer number operates as a built‐in structural variable that programs local coordination and, consequently, the temporal function of the material.

### 2.2 Photophysical Properties of Self‐Trapped Exciton Emission

The photophysics of the Sb‐doped RP NCs are governed by efficient STE emission arising from strong electron‐phonon coupling within the distorted inorganic lattice. Undoped *n* = 1 and *n* = 2 NCs show broad and relatively weak PL centered near 470 and 505 nm, respectively, with large Stokes shifts that are characteristic of STE recombination in low‐dimensional halides (Figure S9). Upon Sb incorporation, the emission intensity and quantum efficiency increase markedly, with the optimum response obtained at 7% Sb (Figure S10). At this composition, the photoluminescence quantum yields (PLQY) reach 65.49% for *n =* 1:Sb and 79.68% for *n =* 2:Sb (Figure S11), indicating that the dopant not only introduces emissive centers but also stabilizes the radiative STE pathway. The broad emission centered near 500 nm is retained in both systems, showing that the essential recombination channel remains the same even though its efficiency is strongly improved by the Sb‐perturbed local environment.

Time‐resolved PL measurements further reveal that the average decay lifetime increases from 4.32 µs for *n* = 1:Sb to 9.97 µs for *n* = 2:Sb, indicating a more stable emissive configuration and reduced non‐radiative loss in the *n* = 2 framework (Figure 2b and Figure S12). Temperature‐dependent spectral analysis provides additional insight into this difference. The larger Huang–Rhys factor extracted for *n* = 2:Sb indicates stronger electron‐phonon coupling and more pronounced lattice relaxation around the excited state [42, 43], while Arrhenius analysis of the integrated emission yields exciton binding energies of 35.3 ± 3.7 meV for *n* = 1:Sb and 43.4 ± 3.1 meV for *n* = 2:Sb (Figures S13 and S14). Together, these results suggest that the *n* = 2 lattice more effectively supports exciton localization and suppresses rapid thermal dissociation. The stronger coupling in the *n* = 2 phase is also consistent with the more asymmetric local coordination inferred from EXAFS, indicating that layer‐dependent structural distortion affects not only trap formation but also the nature of the emissive excited state itself.

**FIGURE 2:**
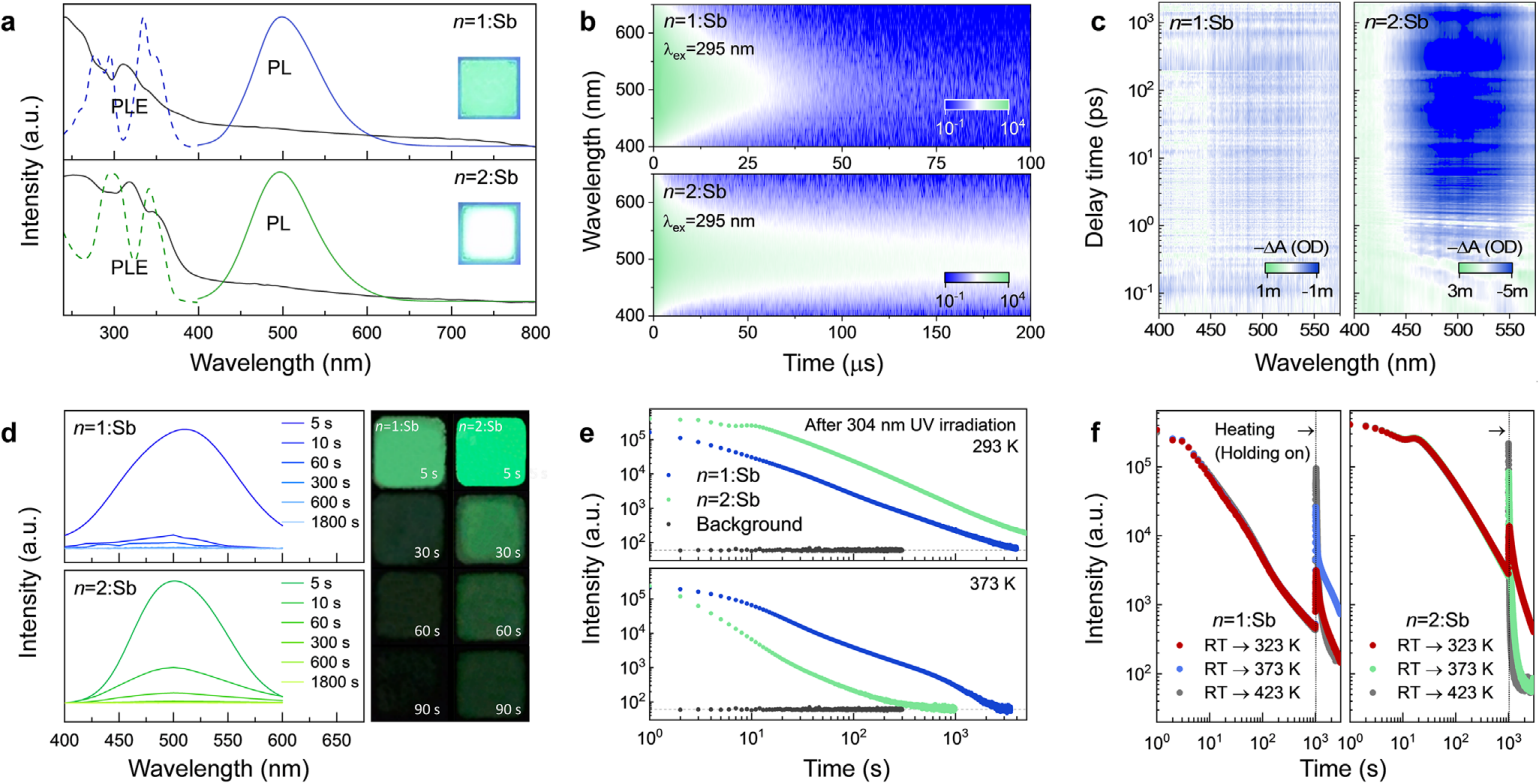
Photophysical Properties of *n* = 1:Sb and *n* = 2:Sb Perovskite NCs. (a) Absorption (gray lines), photoluminescence (PL), and photoluminescence excitation (PLE) spectra for *n* = 1:0.07Sb (λ_ex_ = 295 nm, λ_em_ = 503 nm) and *n* = 2:0.07Sb (λ_ex_ = 295 nm, λ_em_ = 499 nm). Insets: Photographs under 304 nm UV illumination. (b) Time‐resolved PL spectra (pseudo‐color map) under 295 nm excitation. (c) Pseudo‐color map of TA spectra for *n* = 1:0.07Sb and *n* = 2:0.07Sb under 350 nm excitation. (d) Persistent luminescence spectra at room temperature (RT). Insets: Afterglow photographs at indicated delay times. (e) Persistent luminescence decay curves at 293 and 373 K. Gray dots indicate noise level. (f) Thermostimulated persistent luminescence decay: NC films were excited at 304 nm and held at RT for 1000 s before being rapidly heated to the indicated temperatures.

Ultrafast transient absorption (TA) spectroscopy directly probes the excited‐state evolution responsible for this behavior (Figure 2c). After 350 nm excitation, both materials exhibit a broad photoinduced absorption band centered near 500 nm, which is assigned to the excited STE state associated with the distorted Sb‐containing coordination environment. The signal in *n* = 2:Sb rises rapidly and remains substantially stronger over nanosecond timescales than that in *n* = 1:Sb, consistent with more stable exciton trapping and longer‐lived excited‐state population (Figure S15). First‐principles electronic‐structure calculations support this interpretation by showing that Sb‐derived states hybridize with the host bands and generate localized electronic configurations that favor STE formation (Figures S16 and S17). The combined optical and theoretical results therefore establish the *n* = 2 phase as the more effective emissive and storage‐active framework, providing a key rationale for its superior performance in the persistent‐luminescence and device measurements discussed below.

### 2.3 Nanoscale Persistent Luminescence and Programmable Trap Dynamics

A central result of this work is the realization of room‐temperature persistent luminescence in ligand‐free RP perovskite NC films. After cessation of 310 nm excitation, both *n* = 1:Sb and *n* = 2:Sb films exhibit visible afterglow, and the delayed‐emission spectra closely match the steady‐state PL spectra, confirming that the persistent luminescence originates from the same Sb‐perturbed STE recombination channel responsible for prompt emission (Figure 2d and Figure S18). The ability to preserve this mechanism in a ligand‐free NC film is notable because nanoscale perovskite systems typically suffer strong surface‐related losses that shorten carrier‐storage lifetimes. The delayed emission therefore indicates that non‐radiative surface processes do not overwhelm the trap‐mediated storage pathway in the present RP NC system.

The decay kinetics differ substantially between the two RP phases. At 293 K, the afterglow of *n* = 1:Sb decreases to the noise level after about 3000 s, whereas *n* = 2:Sb retains a measurable signal for more than 5000 s, At an elevated temperature of 373 K, the afterglow of *n* = 2:Sb decays rapidly within 1000 s, while that of *n* = 1:Sb persists longer, hinting at different thermal detrapping barriers. This places the system among the longest‐lived afterglow responses reported for nanoscale perovskite systems (Table S5). Thermal stimulation experiments support the interpretation that this behavior is trap‐mediated: after partial dark decay, heating the films triggers a pronounced release of stored carriers and a corresponding increase in emission intensity (Figure 2f). This behavior is consistent with thermally activated release of trapped carriers and agrees well with simulated enhancement factors (Supplementary Note 1). To rule out the effect of temperature‐dependent PL, steady‐state PL spectra were recorded at the same target temperatures (Figure S19). The PL intensity decreases with increasing temperature, opposite to the sharp increase in persistent luminescence upon heating, confirming that the enhancement arises from detrapping rather than radiative efficiency changes. The opposite temperature dependence of the two phases further indicates that the relevant traps differ not only in depth but also in the efficiency with which they can repopulate the emissive state under operating conditions. In other words, the longer afterglow of *n* = 2:Sb does not simply reflect a larger number of traps, but a more favorable balance between carrier capture, storage stability, and delayed radiative release.

To quantify the trap landscape, we performed thermoluminescence (TL) measurements and analyzed the glow curves using Gaussian deconvolution together with variable‐heating‐rate analysis (Figure 3a–d and Figure S20). The dominant effective trap depth is 0.44 ± 0.03 eV for *n* = 1:Sb and 0.35 ± 0.01 eV for *n* = 2:Sb (Supplementary Note 2). Moreover, quantitative TL integral analysis reveals that the trap density of *n* = 2:Sb is approximately 1.64 times higher than that of *n* = 1:Sb (Figure S21), indicating that the RP layer number serves as an effective structural handle for modulating both trap depth and capacity, thereby governing carrier storage and release kinetics. In addition, near‐infrared stimulation can trigger release of stored carriers and enhance the afterglow (Figure S22), confirming the presence of optically addressable trapped states. These observations establish that the two RP frameworks are not merely different emitters, but distinct optical‐memory media in which structural dimensionality dictates the temporal profile of carrier retention. The results further show that deep‐trap engineering in perovskite NCs can be achieved without sacrificing solution processability or the possibility of electrical integration.

**FIGURE 3:**
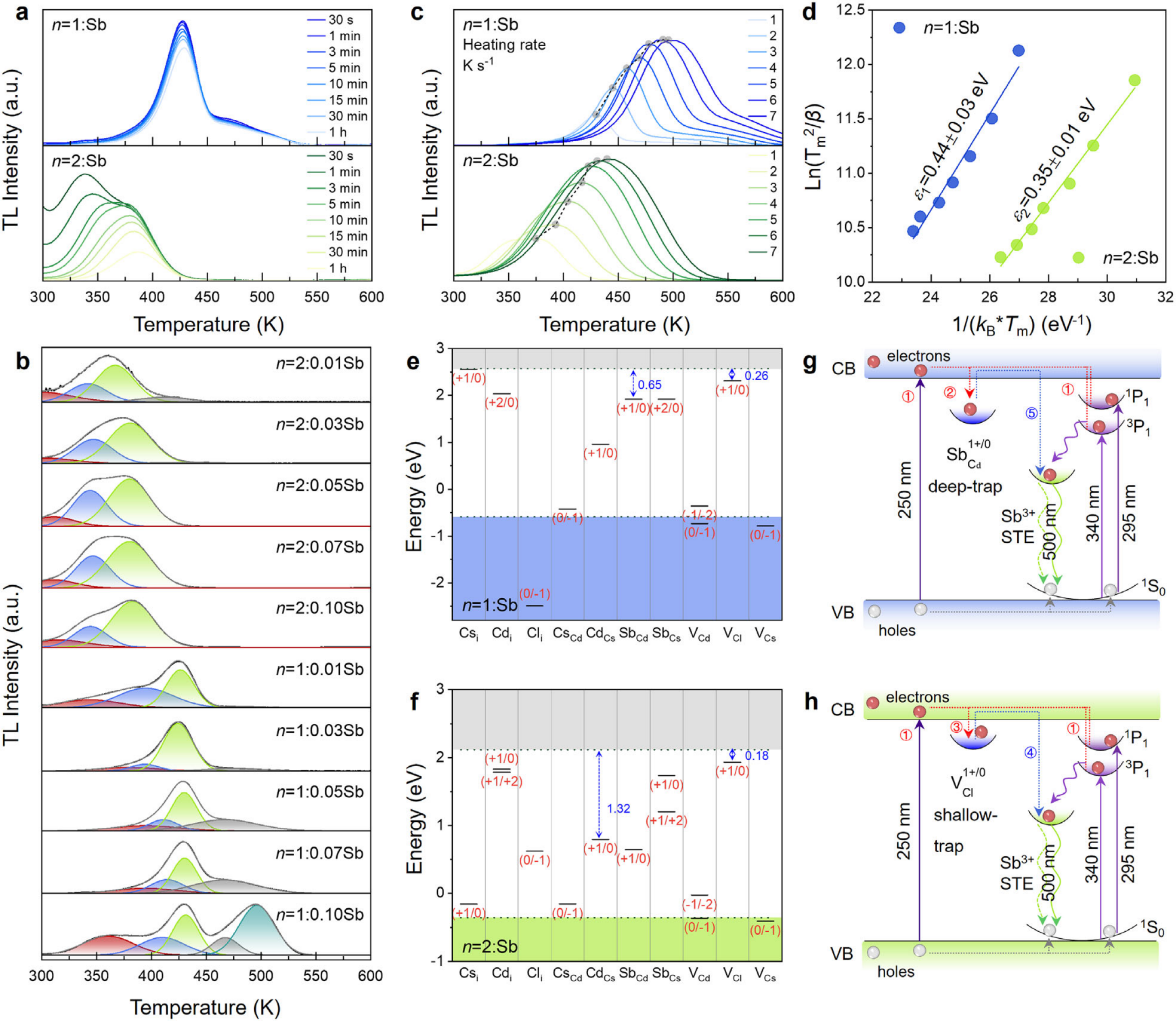
Trap‐State Characterization for *n* = 1:Sb and *n* = 2:Sb NCs. (a) Thermoluminescence (TL) glow curves after 304 nm UV excitation and dark storage for durations ranging from 30 s to 1 h (heating rate: 1 K s^−1^). (b) TL glow curves for *n* = 1:*x*Sb (*x* = 0.01–0.10) and *n* = 2:*y*Sb (*y* = 0.01–0.10). Curves are deconvoluted into bands A1–A5 (shaded regions). (c) TL curves for *n* = 1:0.07Sb and *n* = 2:0.07Sb measured at different heating rates (1–7 K s^−1^). Samples were pre‐irradiated (304 nm, 60 s) and decayed for 180 s before each measurement. (d) Trap‐depth estimation via the Hoogenstraaten method. Thermodynamic charge‐transition levels of point defects in (e) *n* = 1:0.07Sb and (f) *n* = 2:0.07Sb. Blue values (eV) indicate the trap depth relative to the host valence band maximum. Proposed persistent luminescence mechanisms for (g) *n* = 1:0.07Sb and (h) *n* = 2:0.07Sb.

### 2.4 Defect Chemistry and Mechanistic Model of Persistent Luminescence

To understand the origin of the programmable afterglow behavior, we combined TL analysis with first‐principles calculations of defect formation energies. The TL spectra of both materials are broad and can be deconvoluted into multiple trap components, but the dominant peak shifts from approximately 430 K in *n* = 1:Sb to about 375 K in *n* = 2:Sb (Figure 3b). This trend is consistent with the shallower prevailing trap extracted from the Hoogenstraaten analysis and demonstrates that the RP framework directly influences the energy barrier for carrier release. The coexistence of several fitted components also suggests that the stored‐charge process is distributed across a defect ensemble rather than a single isolated state, as is often the case in persistent‐luminescent materials. Nevertheless, the shift in the dominant TL component provides a clear experimental signature of layer‐dependent trap modulation.

Density functional theory (DFT) calculations indicate that the favorable defect species depend strongly on local structure and chemical environment. The calculated energies for various point defects (vacancies, interstitials, antisites) in both *n* = 1:Sb and *n* = 2:Sb are plotted as a function of Fermi level under Cd‐rich and Cd‐poor chemical potentials (Figure S23). For *n* = 1:Sb, synthesized under Cd‐poor conditions, antimony‐on‐cadmium antisites (Sb_Cd_) are energetically favorable and introduce a relatively deep donor level (0.65 eV) that is consistent with the experimentally observed trap depth (Figure 3e). In *n* = 2:Sb, synthesized under Cd‐rich conditions, chlorine vacancies (V_Cl_) become more favorable and generate a shallower electron trap (0.18 eV) (Figure 3f). Although the real NC ensemble is necessarily more complex than an idealized single‐defect model, the calculations and measurements converge on the same physical picture: changing the RP layer number alters the local coordination field, shifts the defect landscape, and redistributes the dominant carrier‐storage sites. This agreement gives mechanistic support to the concept of trap programmability advanced in this work. It also explains why the *n* = 2 phase combines extended afterglow with more efficient delayed recombination, whereas the deeper traps in *n* = 1:Sb favor stronger retention but less effective repopulation of the emissive state at RT.

On the basis of these results, we propose a charging‐storage‐release model for the persistent‐luminescence process (Figure 3g,h). Under ultraviolet excitation, electrons are promoted to the conduction band and captured by metastable defects, while holes remain associated with Sb‐related emissive states. During the dark‐storage period, thermal or optical stimulation gradually releases the trapped electrons, which then repopulate the emissive STE state and generate delayed luminescence. The reversibility of this cycle over repeated operation, together with the stable TL response observed over multiple stimulation cycles (Figure S24), indicates that the trap‐engineered memory process is robust. Importantly, this mechanism directly links local structural distortion to defect chemistry, and defect chemistry to the macroscopic optical‐memory lifetime. It therefore offers a practical framework for designing persistent‐luminescent nanomaterials in which temporal response is an explicit target of structural engineering.

### 2.5 UV‐Sensitive Photonic Synapses and Persistent Optoelectronic Memory

The schematic of the human visual system (Figure 4a) illustrates how light detection, preprocessing, and short‐term memory are seamlessly integrated to process visual information, serving as an analogy for the functionality of an artificial synapse. To translate the intrinsic optical‐memory behavior of the NCs into device function, we fabricated an in‐plane *n* = 2:Sb/SWCNT heterostructure (Figure 4b and Figure S25). In this architecture, the SWCNT network was prepared by conjugated‐polymer‐mediated sorting (Figure S26). SEM and AFM (Figures S27 and S28) show that large‐area, smooth SWCNT films can be deposited on Si wafers to high‐mobility transport and store charge. Meanwhile, the ligand‐free RP NC film acts as the ultraviolet‐sensitive storage layer. Ultraviolet photoelectron spectroscopy (UPS) and optical absorption measurements indicate a type‐II staggered band alignment at the interface (Figure 4c and Figure S29), which favors spatial separation of photogenerated carriers: holes are transferred into the SWCNT network, whereas electrons are retained in the trap‐rich perovskite layer.

**FIGURE 4:**
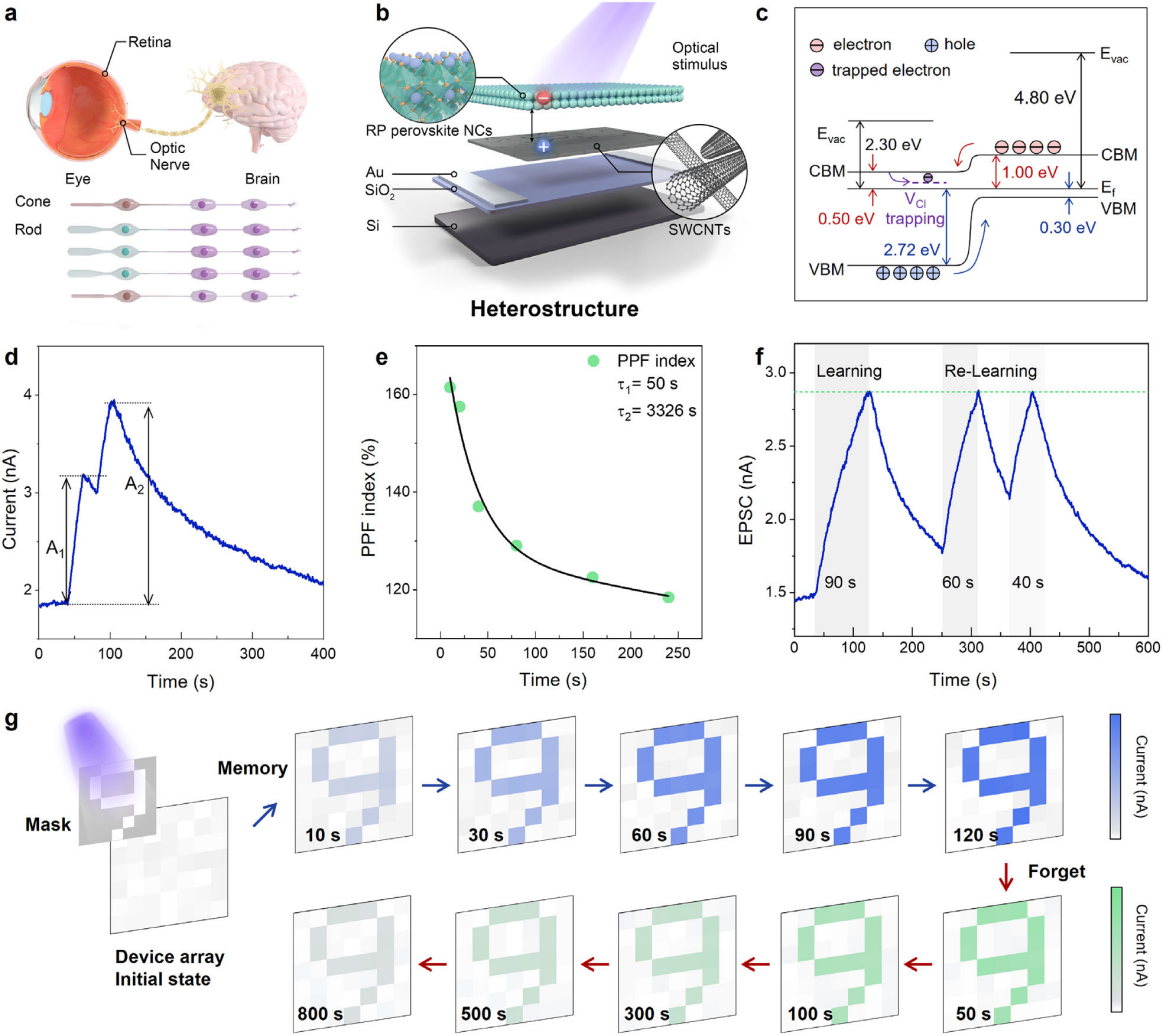
Optoelectronic and Synaptic Characteristics of the *n* = 2:Sb/SWCNT Heterostructure Device. (a) Schematic of the human visual system for analogy. (b) Device architecture of the in‐plane *n* = 2:Sb/SWCNT heterostructure. (c) Energy band diagram of the heterojunction (E_vac_: vacuum level, E_f_: Fermi level). (d) Excitatory postsynaptic current (EPSC) induced by two consecutive 310 nm light pulses (100 µW cm^−2^, 20 s duration). (e) PPF index as a function of pulse interval. (f) Learning–forgetting–relearning behavior under 310 nm illumination (80 µW cm^−2^). (g) Pattern retention: Current response map of a 7×7 pixel array programmed with a UV “9” pattern, showing initial, memorized, and retained states after stimulus removal.

Persistent photocurrent (PPC) is governed by carrier population and release kinetics in defect traps, as probed by TL and PersL, which serve as an optical readout of trap density and synaptic weight. Furthermore, enhancing the radiative efficiency of STE enables optical detection and promotes photocurrent generation [21]. The choice of the *n* = 2 phase is motivated by its combination of high PL efficiency, long afterglow duration, and comparatively favorable carrier‐release kinetics under ambient conditions. The persistent photocurrent originates from Sb‐induced trap states in the *n* = 2:Sb layer, as confirmed by the absence of any long‐lived response in bare SWCNT and undoped *n* = 2/SWCNT devices (Figure S30) [13]. Doping‐dependent photocurrent results further correlate the enhanced PPC and retention with stronger PersL, establishing a direct link between trap density and synaptic response (Figure S31). This heterojunction design converts the intrinsic optical‐memory process of the NCs into a persistent electrical signal that can be read out under a small bias, and the signal can be tuned by the wavelength and intensity of UV light (Figures S32 and S33).

To investigate the device stability, we evaluated its repeatability, environmental resilience, and structural integrity under operational conditions (Figure S34). The *n* = 2:Sb/SWCNT device exhibits stable photoresponse over 30 repeated measurement cycles and maintains 85% of its initial performance after 2 months of N_2_ storage. XRD analysis further confirms that the perovskite NC film retains its structural integrity under prolonged UV irradiation, validating its suitability for long‐term optoelectronic memory applications.

Excitatory postsynaptic currents (EPSCs), which are transient postsynaptic currents induced by presynaptic stimulation, reflect the updating of synaptic weights [44, 45, 46]. Paired‐pulse facilitation (PPF) is a typical biological synaptic behavior for recognizing pulse‐encoded information, in which consecutive presynaptic stimuli can sustainably enhance postsynaptic conductance (synaptic weights). As shown in Figure 4d, when two consecutive 310 nm UV pulses (20 s duration, 100 µW cm^−2^) are applied with a time interval Δ*t*, the EPSC evoked by the second pulse (A_2_) exceeds that of the first (A_1_). The PPF index, defined as (A_2_/A_1_) × 100%, decays with increasing Δ*t* (Figure 4e) and is well fitted by a double‐exponential function:

(1) $$\mathrm{PPFi}\mathrm{ndex}=C_{0}+C_{1}\exp\left(-\frac{\Delta t}{\tau_{1}}\right)+C_{2}\exp\left(-\frac{\Delta t}{\tau_{2}}\right)$$

Where *C_0_*, *C_1_*, and *C_2_* are facilitation constants, the maximum PPF index is 160%, and *τ_1_* and *τ_2_* are two characteristic relaxation times (50 and 3326 s, respectively) for the rapid and slow phases, respectively. These values are analogous to the time scales observed in biological synapses [47, 48]. The coexistence of fast and slow relaxation channels provides a useful analogue to short‐term and longer‐lived synaptic responses, while the persistence of the electrical signal confirms that the NC layer can function as a memory‐active component rather than a purely transient photoabsorber [49, 50]. In this respect, the optoelectronic behavior of the heterostructure directly mirrors the multiscale carrier‐storage dynamics identified in the optical spectroscopy and TL measurements.

Beyond PPF, the heterostructure displays learning‐forgetting‐relearning behavior under repeated ultraviolet stimulation (Figure 4f and Figure S35) [51, 52]. The conductance can be rebuilt more rapidly in successive training cycles, indicating that residual trapped carriers and interfacial charge separation facilitate reactivation of the synaptic state. We further fabricated a 7 × 7 array of photonic synapses and used patterned ultraviolet illumination to write a conductance image corresponding to the numeral “9” (Figure 4g). The pattern remains clearly recognizable for more than 800 s after the stimulus is removed, demonstrating non‐volatile optoelectronic retention at the array level. Beyond the array proof‐of‐concept, the solution‐processable and CMOS‐compatible nature of our platform suggests clear potential for scalable fabrication toward large‐area optoelectronic synaptic arrays [53, 54, 55]. These experiments show that the trap‐programmed RP NCs can support not only long‐lived optical emission, but also persistent photonic‐synapse behavior in an integrated device architecture. More broadly, they illustrate how materials‐level control over trap energetics can be translated into spatially addressable and temporally persistent device function.

## 3 Conclusions

In summary, we show that the trap landscape of Sb‐doped RP perovskite NCs can be modulated through layer‐controlled octahedral distortion, providing a structural route to long‐lived carrier storage and persistent luminescence. The *n* = 1 and *n* = 2 frameworks exhibit distinct local coordination environments and favor different dominant trap states, leading to effective trap depths of 0.44 and 0.35 eV, respectively. This layer‐dependent trap modulation enables room‐temperature afterglow extending beyond 5000 s in the optimized *n* = 2 NC films. By integrating these ligand‐free NCs with semiconducting SWCNT networks, we further realize UV‐sensitive photonic synapses that show persistent photocurrent, PPF, learning‐forgetting‐relearning behavior, and image‐pattern retention. Beyond establishing a high‐performance materials system, this work clarifies how crystallographic dimensionality, local distortion, and defect chemistry can be coupled to program optical‐memory duration in perovskite NCs. The resulting solution‐processed platform provides a useful design principle for memory‐enabled photonic and adaptive optoelectronic devices in which persistent carrier storage is an active functional element rather than an incidental material property. These findings also highlight RP NCs as a versatile testbed for connecting local structure, excited‐state dynamics, and device‐level temporal response within a single materials framework.

## 4 Methods

### 4.1 Materials

Cesium chloride (CsCl, 99.99%), cadmium chloride (CdCl_2_, 99.99%), antimony trichloride (SbCl_3_, 99.9%), isopropyl alcohol (IPA, ≥99.5%), oleic acid (OA, 90%), and dimethyl sulfoxide (DMSO, ≥99.9%) were purchased from Aladdin. Raw arc‐discharge carbon nanotubes (AP‐SWCNTs, ≥60%) were purchased from Carbon Solutions Inc. The dispersant 9‐(1‐octylonoyl)‐9H‐carbazole‐2,7‐diyl (PCz) was synthesized via Suzuki polycondensation in relatively high yield. All materials were used as received without further purification.

#### 4.1.1 Synthesis of Sb^3+^‐Doped Cs_2_CdCl_4_ NCs (n = 1:Sb)

CsCl (0.0561 g) and CdCl_2_ (0.0182 g) were dissolved in 5 mL of DMSO. Separately, SbCl_3_ (0.0810 g) was dissolved in 2 mL of DMSO at RT to form a clear solution. Defined volumes of this SbCl_3_‐DMSO solution (9 µL for 1% Sb, 28 µL for 3% Sb, 46 µL for 5% Sb, 65 µL for 7% Sb, and 93 µL for 10% Sb) were added dropwise to the perovskite precursor solution to achieve the desired doping levels. The mixture was vigorously stirred at RT for 12 h. Subsequently, 500 µL of the precursor solution was rapidly injected into 25 mL of pre‐cooled IPA (2–8°C) under vigorous stirring, inducing immediate NC nucleation. The resulting suspension was centrifuged at 5000 rpm for 5 min, the supernatant was discarded, and the precipitate was collected and redispersed in 250 µL of fresh IPA. Films for optical measurements were prepared by spin‐coating or drop‐casting the dispersion onto a substrate, allowing the solvent to evaporate rapidly at RT.

#### 4.1.2 Synthesis of Sb^3+^‐Doped Cs_3_Cd_2_Cl_7_ NCs (n = 2:Sb)

CsCl (0.0841 g) and CdCl_2_ (0.0610 g) were dissolved in 5 mL of DMSO. Separately, SbCl_3_ (0.0810 g) was dissolved in 2 mL of DMSO at RT. Defined volumes of this SbCl_3_‐DMSO solution (9 µL for 1% Sb, 28 µL for 3% Sb, 46 µL for 5% Sb, 65 µL for 7% Sb, and 93 µL for 10% Sb) were added dropwise to the perovskite precursor solution. The mixture was vigorously stirred at RT for 12 h. Subsequently, 1000 µL of the precursor solution was rapidly injected into 25 mL of pre‐cooled IPA (2–8°C) under vigorous stirring. The resulting suspension was centrifuged at 5000 rpm for 5 min, the supernatant was discarded, and the precipitate was collected and redispersed in 250 µL of fresh IPA. NC films were obtained by spin‐coating or drop‐casting the dispersion onto a substrate, followed by rapid solvent evaporation at RT.

### 4.2 Sample Characterization

X‐ray diffraction (XRD) patterns were collected on a Rigaku SmartLab diffractometer using monochromated Cu Kα radiation (λ = 1.54 Å) over a 2θ range of 10–90°. The XRD data were refined via the Rietveld method using the GSAS‐II program. Transmission electron microscopy (TEM) and scanning transmission electron microscopy (STEM) were performed on a JEM‐2100 electron microscope and a JEM‐2100F field‐emission electron microscope at acceleration voltages of 100 and 200 kV, respectively. Scanning electron microscopy (SEM) images were acquired using a JEOL field‐emission SEM. Ultraviolet photoelectron spectroscopy (UPS) was measured with a Thermo Scientific Nexsa instrument. Sb K‐edge X‐ray absorption spectroscopy (XAS) was performed at the BL14W1 beamline of the Shanghai Synchrotron Radiation Facility (SSRF) using a Si (311) monochromator.

### 4.3 Optical Spectroscopy

Transient absorption (TA) spectroscopy was conducted using an amplified Ti:sapphire femtosecond laser system (Coherent Libra; central wavelength 800 nm, pulse width 50 fs, repetition rate 1 kHz) coupled with an optical parametric amplifier (OPerA Solo) for tunable pump wavelengths (UV to IR) and a Helios pump‐probe setup (Ultrafast Systems) for tunable probe wavelengths (UV to NIR). Raman spectra were acquired using a WITEC alpha300 R confocal Raman microscope. Photoluminescence excitation (PLE), photoluminescence (PL), persistent luminescence spectra, and PL decay curves were recorded on an Edinburgh FLS‐1000 spectrometer. All persistent luminescence decay measurements were performed under identical excitation conditions: a 304 nm UV LED with a power density of 100 µW cm^−2^ and an illumination duration of 60 s, with a fixed sample‐to‐detector geometry. Thermoluminescence (TL) and persistent luminescence decay curves were collected using a Hitachi F‐7000 fluorescence spectrophotometer. X‐ray photoelectron spectroscopy (XPS) was performed with a Thermo Scientific Nexsa XPS system. Heating protocol for thermostimulated persistent luminescence: After 60 s of UV irradiation (304 nm), the natural decay at room temperature was recorded for 1000 s. The temperature was then rapidly raised at a heating rate of 50°C s^−1^ to target temperatures of 323 K, 373 K, and 423 K (requiring approximately 0.5, 1.5, and 2.5 s, respectively). Persistent luminescence decay was recorded while the temperature was held constant.

### 4.4 Device Fabrication

A silicon wafer with a 300 nm SiO_2_ layer was used as the substrate. The substrate was ultrasonically cleaned in acetone, ethanol, and deionized water (15 min each), followed by UV‐ozone treatment for 10 min. A semiconducting SWCNT film was deposited by immersing the substrate in an SWCNT dispersion for 24 h, followed by heating at 120°C for 15 min. Electrodes (5 nm Ti / 45 nm Au, 6 µm channel) were patterned using standard photolithography, electron‐beam evaporation, and lift‐off processes. Finally, the *n* = 2:Sb NC dispersion (250 µL) was mixed with 5 µL OA and spin‐coated onto the patterned substrate at 500 rpm for 10 s and 1000 rpm for 30 s. The device was annealed at 150°C for 30 min to complete the fabrication.

### 4.5 Device Characterization

The electrical characteristics of the device were measured using a Lake Shore probe station coupled with a Keithley 4200‐SCS semiconductor analyzer at a readout bias of 0.5 V. Optical stimulation was provided by Thorlabs LEDs of various wavelengths. All measurements were performed in air at RT.

### 4.6 DFT Calculation

All first‐principles calculations were performed using DFT as implemented in the Vienna Ab initio Simulation Package (VASP). The exchange–correlation energy was treated within the GGA‐PBE functional and the projector augmented‐wave (PAW) method was used to describe core–valence interactions. A plane‐wave cutoff energy of 500 eV and a 3 × 2 × 2 Monkhorst–Pack k‐point grid were adopted for structural optimization. All structures were relaxed until the Hellmann–Feynman forces were below 0.03 eV Å^−1^ and the total energy change was less than 10^−6^ eV. Long‐range van der Waals interactions were included using the DFT‐D3 scheme. For supercell defect calculations, finite‐size effects were corrected by potential alignment and image‐charge corrections, using a static dielectric constant of 6.5.

The formation energy *∆H* of point defects was calculated according to the following equation:

(2) $$\Delta H=\left(E_{D}-E_{H}\right)\;-\sum_{i}n_{i}\left(\mu_{i}+\mu_{i}^{bulk}\right)+q\left(\varepsilon_{VBM}+\varepsilon_{f}\right)+\Delta E_{corr}$$

Where *E_D_* is the total energy of the supercell containing the defect, and *E_H_* is the total energy of the corresponding defect‐free supercell. *n_i_* is the difference in the number of atoms of species *i* between the defect and host supercells, and *µ_i_* is the chemical potential of species *i*, referenced to its bulk value $\mu_{i}^{\mathrm{bulk}}$. *ε_VBM_* is the valence band maximum (VBM) of the host material, and *ε_f_* is the Fermi level measured relative to the VBM.

## Author Contributions

**Xuanyu Zhu**: investigation, writing – original draft, data curation, formal analysis. **Qi Liu**: investigation. **Qi Wei**: investigation. **Hui Ren**: investigation. **Luwei Zhou**: investigation. **Jiangbo Peng**: investigation. **Yuxuan Chen**: investigation. **Jiatong Wang**: investigation. **Leyi Zhao**: investigation. **Pengzhi Wang**: investigation. **Xue Hao**: investigation. **Ting Wang**: investigation, methodology. **Mingjie Li**: conceptualization, supervision, resources.

## Conflicts of Interest

The authors declare no conflicts of interest.

## Supporting information




**Supporting File**: advs78101‐sup‐0001‐SuppMat.docx

## Data Availability

The data that support the findings of this study are available from the corresponding author upon reasonable request.
