# Supplementary material for: Trap‐Programmable Ruddlesden–Popper Perovskite Nanocrystals for Persistent Optoelectronic Memory and Photonic Synapses
Source: Adv Sci (Weinh). 2026 Sep 29:e78101. Online ahead of print. doi: 10.1002/advs.78101 (PMC13624301; doi:10.1002/advs.78101)
Supplement: Supplementary file 1 [file ADVS-9999-e78101-s001.docx]

Supporting Information

Trap-Programmable Ruddlesden–Popper Perovskite Nanocrystals for Persistent Optoelectronic Memory and Photonic Synapses

Xuanyu Zhu, Qi Liu, Qi Wei, Hui Ren, Luwei Zhou, Jiangbo Peng, Yuxuan Chen, Jiatong Wang, Leyi Zhao, Pengzhi Wang, Xue Hao, Ting Wang, Mingjie Li^*^

X. Zhu, Dr. Q. Liu, Dr. Q. Wei, Dr. H. Ren, L. Zhou, Dr. J. Peng, Y. Chen, J. Wang, Dr. L. Zhao, Dr. P. Wang, Prof. M. Li

Prof. M. Li

Department of Physics and Materials, The Hong Kong Polytechnic University, Hung Hom, Kowloon, Hong Kong, China
E-mail: ming-jie.li@polyu.edu.hk

Prof. M. Li
Shenzhen Research Institute, The Hong Kong Polytechnic University, Shenzhen, Guangdong, 518057, China

Prof. M. Li

Photonics Research Institute, The Hong Kong Polytechnic University, Hung Hom, Kowloon, Hong Kong, China

X. Hao, Prof. T. Wang

College of Materials and Chemistry & Chemical Engineering, School of Nuclear Technology and Automation Engineering, Nuclear Technology Key Laboratory of Earth Science, Chengdu University of Technology, Chengdu 610059, China

**Table of contents**

Figure S1 | Schematic of the synthesis and film fabrication process for RP perovskite NCs.

Figure S2 | Cross-section SEM images of Sb-doped NCs.

Figure S3 | HAADF-STEM imaging and EDS mapping of Sb-doped NCs.

Figure S4 | XRD analysis of pristine and Sb-doped NCs films.

Figure S5 | Rietveld refinement of pristine and Sb-doped NCs films.

Figure S6 | Raman spectroscopy of pristine and Sb-doped NCs films.

Figure S7 | XPS of Sb chemical states.

Figure S8 | Sb K-edge EXAFS analysis of local coordination.

Figure S9 | PL and PLE spectroscopy of pristine NCs films.

Figure S10 | PL and PLE spectroscopy as a function of Sb concentration.

Figure S11 | PLQY measurements of Sb-doped NCs films.

Figure S12 | Time-resolved PL spectroscopy of Sb-doped NCs films.

Figure S13 | Temperature-dependent PL spectroscopy (80-300 K) and electro-phonon coupling analysis.

Figure S14 | Arrhenius analysis.

Figure S15 | Photobleaching kinetics analysis.

Figure S16 | First-principles band-structure calculations of pristine and Sb-doped phases.

Figure S17 | PDOS from first-principles calculations.

Figure S18 | Persistent luminescence decay as a function of Sb concentration.

Figure S19 | Temperature-dependent PL spectroscopy (RT, 323 K, 373 K, and 423 K).

Figure S20 | Gaussian-fitted TL intensity.

Figure S21 | TL analysis as a function of Sb concentration.

Figure S22 | NIR--stimulated persistent luminescence measurements of Sb-doped NCs films.

Figure S23 | Defect formation energy calculations under different conditions.

Figure S24 | Thermal cycling stability evaluated by TL.

Figure S25 | Schematic flow for the preparation of *n*=2:Sb/ SWCNTs devices.

Figure S26 | Schematic flowchart for preparing semiconductor SWCNTs.

Figure S27 | SEM image of SWCNTs on the SiO_2_/Si substrate.

Figure S28 | AFM of SWCNT networks.

Figure S29 | Optical absorption and UPS of *n*=2:Sb NCs film.

Figure S30 | Photoresponse measurements of bare SWCNT and undoped *n*=2/SWCNT devices.

Figure S31 | Photoresponse measurements of *n*=2:0.03/SWCNT and *n*=2:0.07/SWCNT devices.

Figure S32 | Photoresponse measurements under different wavelengths of UV illumination.

Figure S33 | Current--time and current--voltage measurements under different intensity of 310 nm UV illumination.

Figure S34 | Stability characterization of the *n*=2:Sb/SWCNT device.

Figure S35 | Conceptual schematic of neuromorphic learning and memory.

Table S1 | Principal parameters of processing and refining of representative samples.

Table S2 | Dopant concentrations in the perovskite NCs with optimal doping concentrations measured by ICP-OES.

Table S3 | Calculated formation energy (*E_form_*) in Sb-doped phases under different occupational schemes.

Table S4 | EXAFS fitting parameters.

Table S5 | Persistent luminescence properties of previously reported perovskite materials.

Supplementary Note 1 | Thermodynamic model for detrapping process.

Supplementary Note 2 | Estimate trap depth.

Supplementary References

**List of Acronyms**

| **Acronyms** | **Definition** |
| --- | --- |
| Cd | Cadmium |
| Cl | Chlorine |
| Cs | Caesium |
| CdCl_2_ | Cadmium chloride |
| CsCl | Caesium chloride |
| CBM | Conduction Band Minimum |
| DFT | Density Functional Theory |
| DMSO | Dimethyl Sulfoxide |
| *E_b_* | Exciton Binding Energy |
| EDS | Energy-Dispersive X-ray Spectroscopy |
| E_f_ | Fermi Energy Level |
| *E_form_* | Formation energy |
| EPSC | Excitatory Postsynaptic Current |
| E_vac_ | Vacuum Energy Level |
| EXAFS | Extended X-Ray Absorption Fine Structure |
| FWHM | Full Width at Half Maximum |
| GGA-PBE | Generalized Gradient Approximation with Perdew |
| HAADF-STEM | High-Angle Annular Dark-Field Scanning Transmission Electron Microscopy |
| HRTEM | High-Resolution Transmission Electron Microscopy |
| ICP-OES | Inductively Coupled Plasma Optical Emission Spectroscopy |
| IPA | Isopropyl Alcohol |
| NCs | Nanocrystals |
| NIR | Near-Infrared |
| *n*=1 | Cs_2_CdCl_4_ |
| *n*=1:Sb | Cs_2_CdCl_4_:0.07Sb |
| *n*=2 | Cs_3_Cd_2_Cl_7_ |
| *n*=2:Sb | Cs_3_Cd_2_Cl_7_:0.07Sb |
| OA | Oleic Acid |
| PAW | Projector Augmented-Wave |
| PDOS | Projected Density of States |
| PL | Photoluminescence |
| PLE | Photoluminescence Excitation |
| PLQY | Photoluminescence Quantum Yield |
| PPF | Paired-Pulse Facilitation |
| RP | Ruddlesden‑Popper |
| RT | Room Temperature |
| SEM | Scanning Electron Microscopy |
| Sb | Antimony |
| Sb_Cd_ | Cation substitution defects (Sb substituting Cd) |
| SbCl_3_ | Antimony trichloride |
| Si | Silicon |
| SiO_2_ | Silicon Dioxide |
| STE | Self-Trapped Exciton |
| STEM | Scanning Transmission Electron Microscopy |
| SWCNTs | Single-Walled Carbon Nanotubes |
| TA | Transient Absorption |
| TEM | Transmission Electron Microscopy |
| TL | Thermoluminescence |
| UPS | Ultraviolet Photoelectron Spectroscopy |
| UV | Ultraviolet |
| VASP | Vienna Ab initio Simulation Package |
| VBM | Valence Band Maximum |
| V_Cd_ | Cadmium Vacancy |
| V_Cl_ | Chlorine Vacancy |
| XPS | X-Ray Photoelectron Spectroscopy |
| XRD | X-Ray Diffraction |

**Figure S1** **| Schematic of the synthesis and film fabrication process for RP perovskite NCs** (Cs*_n_*_+1_Cd*_n_*Cl_3_*_n_*_+1_, *n*=1, 2).

**Figure S2** **| Cross-section SEM images of Sb-doped NCs.** Cross-section SEM images of (a) *n*=1:Sb and (b) *n*=2:Sb films.

**Figure S3** **| HAADF-STEM imaging and EDS mapping of Sb-doped NCs.** High-angle annular dark-field scanning TEM (HAADF-STEM) measurement and EDS mappings of (a) *n*=1:Sb and (b) *n*=2:Sb NCs.

**Figure S4** **| XRD analysis of pristine and Sb-doped NCs films.** XRD patterns of (a) *n*=1, *n*=2, (b) *n*=1:*x*Sb^3+^ (*x*=0.01–0.10) and (c) *n*=2:*y*Sb^3+^ (*y*=0.01–0.10).

**Figure S5** **| Rietveld refinement of pristine and Sb-doped NCs films.** Rietveld structure refinements of (a) *n*=1, (b) *n*=2, (c) *n*=1:0.07Sb^3+^ and (d) *n*=2:0.07Sb^3+^.

**Figure S6** **| Raman spectroscopy of pristine and Sb-doped NCs films.** Raman spectra of *n*=1, *n*=1:0.07Sb, *n*=2 and *n*=2:0.07Sb NCs.

**Figure S7** **| XPS of Sb chemical states.** High-resolution XPS of Sb 3d and O 1s for *n*=1:0.07Sb and *n*=2:0.07Sb NCs.

X‑ray photoelectron spectroscopy (XPS) reveals doublet peaks at binding energies of 531.97/540.77 eV for *n*=1:Sb and 532.35/541.52 eV for *n*=2:Sb, assigned to Sb 3d_5/2_ and Sb 3d_3/2_, respectively

**Figure S8** **| Sb K-edge EXAFS analysis of local coordination.** k^2^-weighted Sb K-edge EXAFS spectra for *n*=1:0.07Sb and *n*=2:0.07Sb NCs.

**Figure S9** **| PL and PLE spectroscopy of pristine NCs films.** PLE and PL spectra of pure (a) *n*=1 and (b) *n*=2 NCs.

**Figure S10** **| PL and PLE spectroscopy as a function of Sb concentration.** PL spectra of *n*=1:*x*Sb (*x*=0.01–0.10) under excitation at (a) 295 nm and (b) 340 nm. PL spectra of *n*=2:*y*Sb (*y*=0.01–0.10) under excitation at (c) 295 nm and (d) 340 nm. PLE spectra of (e) *n*=1:*x*Sb and (f) *n*=2:*y*Sb monitored at 500 nm.

**Figure S11** **| PLQY measurements of Sb-doped NCs films.** PLQY values of (a) *n*=1:0.07Sb and (b) *n*=2:0.07Sb NCs under 295 nm and 335 nm excitation.

**Figure S12** **| Time-resolved PL spectroscopy of Sb-doped NCs films.** Pseudo color map of time-resolved PL spectra of (a) *n*=1:0.07Sb and (b) *n*=2: 0.07Sb under 340 nm excitation. PL decay curves of the emission at (c) 503 nm for *n*=1: 0.07Sb and (d) 499 nm for *n*=2: 0.07Sb.

**Figure S13** **| Temperature-dependent PL spectroscopy (80-300 K) and** **electro-phonon coupling analysis.** (a, b) Pseudocolor contour maps of normalized PL spectra and (c) Plot of the full widths at half maximum (FWHM) of *n*=1:0.07Sb and *n*=2:0.07Sb emissions versus the temperature. Fitting yields the phonon energy (*ħω_phonon_*) and Huang–Rhys factor (*S*).

To quantitatively understand the STE formation, we analyzed the temperature‑dependent PL spectra. In this case, the intensity of electron-phonon coupling significantly defines the formation tendency of STE emissions, which can be assessed using the Huang-Rhys factor (*S*) and the phonon energy (*ħω*_phonon_) via the following equation:

$$\begin{aligned} FWHM=2.36\sqrt{S}\hbar\omega_{\mathrm{phonon}}\sqrt{\coth\frac{\hbar\omega_{\mathrm{phonon}}}{2k_{B}T}}\#\left( 1 \right) \end{aligned}$$

where *ħω*_phonon_, *T*, and *k_B_* denote the longitudinal optical phonon energy, temperature and Boltzmann constant, respectively. Fitting yielded *S* = 17.10 ± 0.9 and *ħω* = 31.2 meV for *n*=1:Sb, and *S* = 19.42 ± 1.0 and *ħω* = 33.5 meV for *n*=2:Sb. The larger *S* factor for *n*=2:Sb signifies stronger electron‑phonon coupling, which facilitates the lattice distortion required for efficient STE formation.

**Figure S14** **| Arrhenius analysis.** (a, b) integrated PL intensity and peak position versus temperature; (c, d) Arrhenius analysis of integrated PL intensity, yielding activation energies of 35.3 ± 3.7 meV (*n*=1:0.07Sb) and 43.4 ± 3.1 meV (*n*=2:0.07Sb).

The temperature‑dependent PL intensity was used to estimate the exciton binding energy (*E_b_*) via the equation:

$$\begin{aligned} I\left( T \right)=\frac{I_{0}}{1+Aexp\left( -\frac{E_{b}}{k_{b}T} \right)}\#\left( 2 \right) \end{aligned}$$

where *I* is the integrated intensity of each PL spectrum. We obtained *E_b_* = 35.3 ± 3.7 meV for *n*=1:Sb and 43.4 ± 3.1 meV for *n*=2:Sb. These values are comparable to the phonon energies, suggesting a scenario where phonon‑assisted relaxation efficiently channels free excitons into the self‑trapped manifold, out‑competing band‑edge recombination.

**Figure S15** **| Photobleaching kinetics analysis.** Photobleaching kinetics at 500 nm of *n*=1:0.07Sb and *n*=2:0.07Sb under 350 nm excitation.

**Figure S16** **| First-principles band-structure calculations of pristine and Sb-doped phases.** The calculated band structure of (a) *n*=1, (b) *n*=2, (c) *n*=1:Sb and (d) *n*=2:Sb.

The calculated bandgap of the undoped *n*=1 host is 3.25 eV (direct), while *n*=2 has a 2.48 eV indirect gap.

**Figure S17** **| PDOS from first-principles calculations.** The Projected Density of States (PDOS) of (a) *n*=1, (b) *n*=2, (c) *n*=1:Sb and (d) *n*=2:Sb.

The conduction band minimum (CBM) and valence band maximum (VBM) are primarily composed of Cd 5s and Cl 3p orbitals, respectively. Upon Sb^3+^ doping, Sb 5s states hybridize with Cl 3p states at the CBM, and Sb 5p states contribute to the VBM, effectively reducing the bandgap to 2.94 eV (*n*=1:Sb) and 2.28 eV (*n*=2:Sb).

**Figure S18 | Persistent luminescence decay as a function of Sb concentration.** Persistent luminescence decay curve of (a) *n*=1:*x*Sb (*x*=0.01–0.10) and (b) *n*=2:*y*Sb (*y*=0.01–0.10) after 304 nm UV excitation for 60 s.

**Figure S19 | Temperature-dependent PL spectroscopy (RT, 323 K, 373 K, and 423 K).** Temperature-dependent PL spectra of (a) *n*=1:Sb and (b) *n*=2:Sb NC films at RT, 323 K, 373 K, and 423 K. (c) Integrated PL intensity versus temperature of *n*=1:Sb and *n*=2:Sb films.

**Figure S20** **| Gaussian-fitted TL intensity.** Fitted TL intensities of different trap components as a function of Sb^3+^ concentration for *n*=1 and *n*=2.

**Figure S21** **| TL analysis as a function of Sb concentration.** TL curves of *n*=1:*x*Sb and *n*=2:*y*Sb (*x*, *y* = 0.01–0.10). Measurements were performed at a heating rate of 1 K s^-1^ after 60 s irradiation with a 304 nm UV lamp and 180 s decay.

In both RP phases, trap concentration peaks at 7% Sb³⁺, indicating that Sb³⁺ doping reshapes intrinsic defects and enhances trap density.

**Figure S22** **| NIR–stimulated** persistent luminescence **measurements of Sb-doped NCs films.** Persistent luminescence decay curves under continuous 980 nm NIR photo-stimulation.

**Figure S23** **| Defect formation energy calculations under different conditions.** Calculated formation energies of point defects in various charge states as a function of the Fermi level in the bandgap for (a) *n*=1 and (b) *n*=2 in Cd-poor and Cd-rich cases.

The calculated energies for various point defects (vacancies, interstitials, antisites) in both *n*=1:Sb and *n*=2:Sb are plotted as a function of Fermi level under Cd‑rich and Cd‑poor chemical potentials. The most thermodynamically favorable defects are strongly influenced by synthesis conditions. For *n*=1:Sb, synthesized under Cd‑poor conditions, the Sb_Cd_ antisite defect (where Sb^3+^ occupies a Cd^2+^ site, written as Sb_Cd_) has a very low formation energy and introduces a deep donor level at ~0.65 eV below the CBM. In *n*=2:Sb, synthesized under Cd‑rich conditions, the chlorine vacancy (V_Cl_) is the dominant defect, creating a shallower electron trap at ~0.18 eV below the CBM.

**Figure S24** **| Thermal cycling stability evaluated by TL.** Thermal stability of *n*=1:0.07Sb and *n*=2:0.07Sb films during 10 charge-discharge cycles (Charge: 60 s irradiation with 304 nm UV light at RT; Discharge: heating from 300 to 600 K). Blue and red dots represent initial intensity and peak TL intensity, respectively.

The charging and emission process was reproducible over more than ten UV irradiation–heating cycles, indicating excellent stability and reversibility.

**Figure S25** **| Schematic flow for the preparation of *n***=**2:Sb/ SWCNTs devices.**

A semiconducting SWCNT film was formed by immersing a UV–ozone plasma-treated SiO_2_/Si substrate in a SWCNT solution for 24 h, followed by thermal treatment at 120 °C for 15 min. Electrode deposition (45 nm Au/5 nm Ti) was achieved by photolithography, electron-beam evaporation, and lift-off. The *n*=2:Sb film was spin-coated at 500 rpm for 10 s and 1000 rpm for 30 s, then annealed at 150 °C for 30 min to complete the device.

**Figure S26** **| Schematic flowchart for preparing semiconductor SWCNTs.**

AP-SWNTs have a typical diameter distribution with a peak value of 1.55 ± 0.1 nm and a bundle length of 1-5 µm. High-purity semiconducting SWNTs are sorted by mixing dispersant PCz (9-(1-octylonoyl)-9H-carbazole-2,7-diyl, 5 mg) with AP-SWNTs (5 mg) in 20 mL toluene, followed by sonication for 1 h, and then centrifugation at 15,000 g for 1 h to remove CNT bundles and insoluble substances. Finally, the supernatants were collected and re-diluted in chloroform.

**Figure S27** **| SEM image of SWCNTs on the SiO_2_/Si substrate** (scale bar, 20 µm).

Scanning electron microscope (SEM) images of SWCNT films on the SiO_2_/Si substrate show that the SWCNTs are randomly distributed within the network.

**Figure S28** **| AFM of SWCNT networks.** (a) Two-dimensional image of a SWCNT network. (b) Height data extracted along the red dashed line in (a).

The root-mean-square (RMS) roughness of the SWCNT surface is 0.79 nm, and the film thickness is approximately 6 nm, confirming uniform, high-quality SWCNT networks on the wafer via dip-coating.

**Figure S29** **| Optical absorption and UPS of *n***=**2:Sb NCs film.** (a) Absorption spectrum and (b) Tauc plot of *n*=2:Sb film. UV photoelectron spectroscopy (UPS) of *n*=2:Sb film under (c) -10 V and (d) 0 V.

The bandgap of *n*=2:Sb is ~ 3.27 eV. From the UPS spectrum, cut-off energy (E_cut-off_) and onset energy (E_onset_) were determined to be 18.92 eV and 2.72 eV, respectively. The work function (W_F_) is 2.30 eV, calculated from W_F_ = 21.22 eV - E_cut-off_. The VBM of *n*=2:Sb is -5.02 eV, calculated from E_VB_ = -(W_F_ + E_onset_).

**Figure S30 | Photoresponse measurements of bare SWCNT and undoped *n*=2/SWCNT devices.** I-t curve of (a) bare SWCNT and (b) undoped *n*=2/SWCNT devices (310 nm, 100 µW cm^-2^).

The bare SWCNT device exhibited no photoresponse to 310 nm UV light, with its current remaining constant at ~1.05 pA before and after irradiation. This stems from ultra-fast exciton recombination, which prevents efficient carrier separation and the generation of photocurrent. The undoped *n*=2/SWCNT device saturated within 5 s and decayed to baseline within 30 s upon cessation of UV excitation, indicating that undoped *n*=2 NCs lack the trap states required for long-term carrier storage.

**Figure S31 | Photoresponse measurements of *n*=2:0.03/SWCNT and *n*=2:0.07/SWCNT devices.** I-t curve of *n*=2:0.03/SWCNT and *n*=2:0.07/SWCNT devices (310 nm, 100 µW cm^-2^).

The photocurrent of *n*=2:0.03Sb rises to ~3 nA after 120 s excitation and decays to 1.67 nA (56%) after 100 s cessation. In contrast, the *n*=2:0.07Sb device reaches ~4.96 nA and retains 3.32 nA (67%) after 100 s cessation.

**Figure S32** **| Photoresponse measurements under different wavelengths of UV illumination.** I-t curve of devices under various UV wavelengths (100 µW cm^-2^).

The device shows a photoresponse that decays with increasing wavelength in the 280–395 nm range, consistent with the absorption characteristics of the *n*=2:Sb layer.

**Figure S33 | Current–time and current–voltage measurements under different intensity of 310 nm UV illumination.** (a) I-t and (b) I-V curves under different intensities of 310 nm UV light.

At higher intensities, the device shows a stronger persistent photoresponse and slower photocurrent decay, indicating enhanced carrier trapping and prolonged retention of photogenerated carriers in the heterojunction.

**Figure S34 | Stability characterization of the *n*=2:Sb/SWCNT device.** (a) Histogram of peak photocurrent values recorded from 30 repeated measurement cycles (310 nm UV, 100 µW cm^-2^, 120 s). (b) Photoresponse curves before and after 2 months of N_2_ storage. (c) XRD patterns of the *n*=2:Sb NC film: as-prepared, after 2 months of N_2_ storage, and after 60 mins of continuous UV irradiation.

**Figure S35** **| Conceptual schematic of neuromorphic learning and memory.** Learning, forgetting, relearning, and memory processes in neuromorphic visual systems.

**Table S1** **| Principal parameters of processing and refining of representative samples.**

| Compound | *n*=1 | *n*=1:0.07Sb^3+^ | *n*=2 | *n*=2:0.07Sb^3+^ |
| --- | --- | --- | --- | --- |
| Space group | I4/mmm | | | |
| a=b, Å | 5.25999 | 5.25580 | 5.23481 | 5.23002 |
| c, Å | 16.90323 | 16.89168 | 27.14697 | 27.12808 |
| V, Å^3^ | 467.671 | 466.606 | 743.914 | 742.038 |
| R_wp_, % | 6.76 | 8.85 | 9.03 | 8.71 |
| χ^2^ | 3.12 | 4.15 | 4.23 | 4.15 |

**Table S2 | Dopant concentrations in the perovskite NCs with optimal doping concentrations measured by ICP-OES.**

| Crystal phase | Cd (mmol/L) | Sb (mmol/L) | Sb/Cd (%) |
| --- | --- | --- | --- |
| Cs_2_CdCl_4_:0.07Sb^3+^ | 3.757 | 0.2497 | 6.65% |
| Cs_3_Cd_2_Cl_7_:0.07Sb^3+^ | 4.621 | 0.2846 | 6.16% |

**Table S3** **|** **Calculated formation energy (*E_form_*) in Sb-doped phases under different occupational schemes.**

| Crystal phase | Occupied sites | *E_form_* (eV) |
| --- | --- | --- |
| Cs_2_CdCl_4_:Sb^3+^ | Sb→Cd | -4.18 |
|  | Sb→Cs | -0.04 |
| Cs_3_Cd_2_Cl_7_:Sb^3+^ | Sb→Cd | -6.57 |
|  | Sb→Cs | -0.08 |

**Table S4** **| EXAFS fitting parameters.** (Ѕ_0_^2^ = 0.80)

| Sample  Sb K-edge | CN | Bond | ΔE_0_ (eV) | Reff (Å) | R (Å) | Disorder (σ²) | R factor |
| --- | --- | --- | --- | --- | --- | --- | --- |
| Cs_2_CdCl_4_: Sb | 4 | Sb-Cl_1_ | 8.850±5.276 | 2.630 | 2.476±0.045 | 0.00848±0.00249 | 0.0307759 |
|  | 2 | Sb-Cl_2_ | 8.850±5.276 | 2.515 | 2.202±0.037 | 0.00802±0.00635 |  |
| Cs_3_Cd_2_Cl_7_: Sb | 5 | Sb-Cl_2_ | 2.447±4.885 | 2.627 | 2.508±0.042 | 0.01503±0.00309 | 0.0143155 |
|  | 1 | Sb-Cl_1_ | 2.447±4.885 | 2.589 | 2.154±0.067 | 0.01262±0.00898 |  |

**Table S5** **| Persistent luminescence properties of previously reported perovskite materials.** (Single Crystal, SCs; microcrystals, MCs).

| Perovskites | Morphology | Persistent luminescence peak (nm) | Excitation source | Ambient temperature | Persistent luminescence lifetime | Ref. |
| --- | --- | --- | --- | --- | --- | --- |
| CsCdCl_3_: Mn^2+^ | SCs | 596 nm | UV | RT | 1480 s | ^1^ |
| CsCdCl_3_ | SCs | 580 nm | UV | RT | 6000 s | ^2^ |
| CsCdCl_3_:0.8%Br | SCs | 482 nm/ 595 nm | UV | RT | ＞2000 s | ^3^ |
| CsCdCl_3_:10%Sn | SCs | 595 nm | UV | RT | ＞2000 s |  |
| Cs_2_Na_0.2_Ag_0.8_InCl_6_: Mn^2+^ | SCs | 622 nm | UV | RT | ＞5400 s | ^4^ |
| Cs_2_CdCl_4_: Mn^2+^ | SCs | 612 nm | UV | RT | 600 s | ^5^ |
| Cs_2_CdCl_4_: Sb^3+^ | MCs | 515 nm | UV | RT | 600 s | ^6^ |
| Cs_3_Cd_2_Cl_7_:1%Sb^3+^,1%Mn^2+^ | MCs | 580 nm | UV | RT | ＞3600 s | ^7^ |
| Cs_2_CdCl_4_:7%Sb^3+^ (*n*=1:Sb) | **NCs film** | 500 nm | UV | **373 K** | **＞2500 s** | **This work** |
| Cs_3_Cd_2_Cl_7_:7%Sb^3+^ (*n*=2:Sb) | **NCs film** | 500 nm | UV | RT | **＞5000 s** | **This work** |

**Supplementary Note 1** **| Thermodynamic model for detrapping process.**

In a simplified case, it is assumed that the number of electrons trapped at time *t* is $n_{t}$, and all electrons are located in a trap with a depth of *ε* (in eV). The probability $\alpha_{p}$ of an electron being thermally activated from the trap is defined by the following formula:

$\begin{aligned} \alpha_{p}=s\cdot exp\frac{-\varepsilon}{k_{B}\cdot T}\#\left（ 3 \right） \end{aligned}$ere *s* denotes the frequency factor, representing the number of times per second that electrons are activated ( in s^-1^, *k_B_* is Boltzmann's constant, and *T* is the environmental temperature.

The variation in captured electrons over time *t* is determined by the following formula:

$$\begin{aligned} \frac{dn_{t}}{dt}=n_{t}\cdot\alpha_{p}\#\left( 4 \right) \end{aligned}$$

The intensity of trap-induced persistent luminescence *I* is proportional to the number of electrons released from the trap at time *t*, and may therefore be expressed as:

$\begin{aligned} I\propto\frac{dn_{T}}{dt}=n_{t}\cdot s\cdot exp\frac{-\varepsilon}{k_{B}\cdot T}\#\left( 5 \right) \end{aligned}$en the environmental temperature suddenly changes from T_1_ to T_2_, the intensity of persistent luminescence should have a variation of:

$$\begin{aligned} \frac{I_{2}}{I_{1}}=\frac{exp\frac{-\varepsilon}{k_{B}\cdot T_{2}}}{exp\frac{-\varepsilon}{k_{B}\cdot T_{1}}}\#\left( 6 \right) \end{aligned}$$

Therefore, after transformation, the following relationship is obtained:

$$\begin{aligned} \frac{I_{2}}{I_{1}}=\exp\left[ \frac{\varepsilon}{k_{B}}\cdot\left( \frac{1}{T_{1}}-\frac{1}{T_{2}} \right) \right]\#\left( 7 \right) \end{aligned}$$

Equation (5) reveals that the intensity ratio is increased (*I_2_*/ *I_1_*＞1) upon abrupt temperature elevation (*T_2_*＞*T_1_*), exhibiting an exponential relationship with the difference of the reciprocal of temperature *T_2_*.

If trap depths of 0.44 eV and 0.35 eV are taken (the intermediate values for *n*=1:Sb and *n*=2:Sb based on DFT calculations in Figure 3c), when the temperature is abruptly raised from 293 K (T_1_) to 323, 373, and 423 K (T_2_), the variation in the persistent luminescence intensity I_2_/I_1_ for *n*=1:Sb should be 6.62, 55.30, and 201.18, respectively. For *n*=2:Sb, the variation in the persistent luminescence intensity I_2_/I_1_ should be 4.84, 29.54, and 76.85, respectively. It is noted that the experimental values (5.04, 41.99 and 211.80 for *n*=1:Sb, and 3.62, 19.55 and 70.82 for *n*=2:Sb) in Figure 2f generally agree with the calculated values.

**Supplementary Note 2** **| Estimate trap depth.**

By employing the variable heating rate method (Figure 3c), we can estimate the effective trap depth (*ε*) using the initial rise analysis and the Hoogenstraaten equation:

$$\begin{aligned} \frac{\beta\cdot\varepsilon}{k_{B}\cdot T_{m}^{2}}=s\cdot exp\left( \frac{-\varepsilon}{k_{B}\cdot T_{m}} \right)\#\left( 8 \right) \end{aligned}$$

where *β* is the heating rate, *T*_m_ is the peak temperature, and *s* is the frequency factor. Plotting ln(*T*_m_^2^/*β*) versus 1/(*k*_B_ *T*_m_) yields a linear relationship (Figure 3d), from which we extract *ε*_1_ = 0.44 ± 0.03 eV for *n*=1:Sb and *ε*_2_ = 0.35 ± 0.01 eV for *n*=2:Sb.

**Supplementary References**

1	He, S. *et al.* Highly stable orange‐red long‐persistent luminescent CsCdCl3: Mn2+ perovskite crystal. *Angewandte Chemie International Edition* **61**, e202208937 (2022).

2	Yang, R. *et al.* High‐Efficiency and Stable Long‐Persistent Luminescence from Undoped Cesium Cadmium Chlorine Crystals Induced by Intrinsic Point Defects. *Advanced Science* **10**, 2207331 (2023).

3	Chen, T. & Yan, D. Full-color, time-valve controllable and Janus-type long-persistent luminescence from all-inorganic halide perovskites. *Nature Communications* **15**, 5281 (2024).

4	Zheng, W. *et al.* Solution‐grown chloride perovskite crystal of red afterglow. *Angewandte Chemie* **133**, 24655-24660 (2021).

5	Liu, Y. *et al.* Achieving color‐tunable long persistent luminescence in Cs2CdCl4 Ruddlesden‐Popper phase perovskites. *Angewandte Chemie International Edition* **62**, e202308420 (2023).

6	Liu, Z. *et al.* A memristor-based adaptive neuromorphic decoder for brain–computer interfaces. *Nature Electronics* **8**, 362-372, doi:10.1038/s41928-025-01340-2 (2025).

7	Liu, S. *et al.* Inconsistency Between Photoluminescence and Persistent Luminescence in Sb3+/Mn2+ Co‐Doped Cs3Cd2Cl7. *Laser & Photonics Reviews* **19**, e01241, doi:10.1002/lpor.202501241 (2025).
